# Bacteriology

**Published:** 1905-05-06

**Authors:** 


### BACTERIOLOGY.

**Micro-organisms in Normal Tissues.**—Nicholls [1] finds that micro-organisms can readily be detected in the cells and capillary channels of the mesentery of healthy animals, whence they are carried to the liver, kidneys, and other organs. Adami also observed this vital absorption of bacteria. Nicholls thinks that bacteria are constantly passing from various surfaces of the body to become lodged in the tissues, principally the lymph glands. Ruffer demonstrated bacteria between the epithelial cells of the intestine and within Peyer's patches. Pneumococci and tubercle bacilli have been demonstrated in the peribronchial glands of healthy animals. The epithelial surfaces afford no protection against the entry of bacteria, but such bacteria as find entry are speedily destroyed in the tissues. If these results can be substantiated it shows how important is "individual resistance" as a practical factor in immunity.

**Anthrax.**—The serum treatment of anthrax has only recently been adopted in this country, and two successful cures without excision have been reported during the present year. In both cases the serum used was that prepared by Professor Sclavo. In the first case, reported by Lockwood and Andrewes,[2] the patient, a man, presented himself four days after the appearance of the first papule, and virulent anthrax bacilli were demonstrated in the pustule. The initial dose of the serum was 40 c.cm., and as the local conditions showed no signs of progression, and as the general condition was satisfactory, no further dose was administered. Three days after the injection there was distinct improvement, and no anthrax bacilli could be cultivated from the pustule. The local lesion healed rapidly, leaving a scar resembling that of vaccination. The second case, reported by Bowlby and Andrewes,[3] was detected and treated five days after the appearance of the primary lesion. In this case also a dose of 40 c.cm. of serum was given, and the improvement in the patient's general condition was noted within 24 hours, although for some days the œdema around the slough continued to expand. Cultures of virulent anthrax bacilli were made on admission, but 19 hours after the use of the serum, cultures similarly made proved to be sterile. The œdema around the pustule is due to the breaking up of the bacilli and the release of an intracellular toxin. This method of treatment is peculiarly suitable for cases of anthrax of the face in which removal might lead to considerable disfigurement. Legge,[4] in the Milroy lectures on anthrax, deals with 261 cases notified between 1899 and 1904. Of these 26·1 per cent. occurred in the worsted and wool trade, 24·3 in workers in horsehair and bristles, 24·4 in hides and skins, and 35·3 in other industries. The total mortality amongst the cases was 25·6 per cent. Only six cases of internal anthrax were discovered. No case could be traced to infection from Australian or New Zealand wool. Persian wool was a fruitful source, as also was mohair imported from European and Asiatic Turkey, and horsehair from Russia and China. The incidence of anthrax reaches its maximum in July and August, and this is probably due to considerations of temperature, for below 18°C. and above 42°C. the capacity for sporulation ceases. The spread of anthrax is dependent upon factory and workshop conditions, the use of factory refuse as manure, the presence of factory refuse in sewage, the consumption by cattle of infected fodder, and imported foodstuffs, and upon the use of artificial manure. Legge has collected 67 cases treated by antianthrax serum. The time occupied in the cure varied from eight to 14 days and a noticeable feature was the very slight resultant scarring. Two of the cases ended fatally, but in these treatment had been delayed. The initial dose was generally 40 c.cm. Sclavo collected 164 cases treated by serum with a mortality of 6·09 per cent., as compared with 24·1 per cent. for the whole of Italy. Twelve cases in all of anthrax have been treated by Sclavo's serum in England, but in 10 of these excision of the pustule was first performed. The most powerful serum is obtained by immunising the ass with attenuated cultures of the bacillus anthracis, and this may be used not only for curative but also for protective purposes. The immunity conferred is probably the result of the fabrication by the body cells of bactericidal substances which, passing into the intravascular and extravascular fluids, destroy the bacilli.

**Small pox and Vaccinia.**—De Waele and Sugg [5] have isolated a streptococcus from the blood and vesicles of persons suffering from small-pox, and they have also isolated the organism from the tissues after death. The serum of persons suffering from small-pox gave an agglutinative reaction with these organisms, and also with streptococci found in a variety of calf-vaccine lymphs. The serum of vaccinated calves also agglutinated emulsions of these streptococci whilst other streptococci were unaffected. Korté,[6] examining lymph from small-pox, vaccinia, and varicella in hanging drop preparations, found large numbers of unicellular elements showing amœboid movement. These elements were found in lymph that had been stored for six months and so could not be confounded with leucocytes, which disappear spontaneously from lymph after six days storage. They could not be easily fixed, as can be leucocytes. If fixed with alcohol and ether and stained with methylene blue, the nucleus could be demonstrated in the organism obtained from varicella, but not in that obtained from variola and vaccinia. The organism is a spherical granular body, $\frac{1}{2500}$ of an inch in diameter.

It possesses spores which become free and are mobile. The lymph should be taken from the pock of variola on the fifth day, and from the vaccination papule on the eighth day, as by the tenth day it spontaneously disappears. In varicella the vesicle contents may be examined on the first, second, or third day. Kalkins[7] gives a further description of the organisms of variola and varicella, first described by Guarnieri in 1892. They are to be demonstrated in the skin lesions by methods of differential staining, that recommended being Borrel's indigo carmine and picric acid stain preceded by magenta. The parasite is a sporozoon and may be either intracellular or intranuclear. The intracellular form is reproduced asexually, the intranuclear sexually. The former start as gemmules, have amœboid movement and enter the cells of the skin. In the nuclei of cells gametocytic forms develop, conjugate, and form zygotes and sporoblasts and secondary sporoblasts. The life cycle thus resembles that of the malaria parasite. These parasites, which have been named cytocyetes variolæ, are to be found in lesions in the skin of monkeys inoculated with variola virus. They have also been detected in the lesions caused by inoculation of a rabbit's cornea with variolous material. Leucocytosis is generally present in variola, which develops with the cutaneous lesion and declines with convalescence. The increase is chiefly in the mononuclear leucocytes. In unfavourable cases the leucocyte count is high during the early stages and then declines until death. The parasites of variola and vaccinia cannot be detected in the blood, but Brenckerhoff[8] found them present and virulent in small-pox scabs which had been kept as long as 88 days.

[1] Med. Rec., June 25, 1904. [2] Brit. Med. Jour., Jan. 7, 1905. [3] Ibid., Feb. 11, 1905. [4] Lancet, March 18, 25, April 1, 1905. [5] Arch. Inter. de Phar. et Therap., Vol. XIII., 1904. [6] Prac., Jan. 1905. [7] Jour. Med. Rec., Feb. 1904. [8] Ibid.

